# Radiocarbon Tracers in Toxicology and Medicine: Recent Advances in Technology and Science

**DOI:** 10.3390/toxics7020027

**Published:** 2019-05-09

**Authors:** Michael A. Malfatti, Bruce A. Buchholz, Heather A. Enright, Benjamin J. Stewart, Ted J. Ognibene, A. Daniel McCartt, Gabriela G. Loots, Maike Zimmermann, Tiffany M. Scharadin, George D. Cimino, Brian A. Jonas, Chong-Xian Pan, Graham Bench, Paul T. Henderson, Kenneth W. Turteltaub

**Affiliations:** 1 Biosciences and Biotechnology Division, Lawrence Livermore National Laboratory, Livermore, CA 94550, USA; malfatti1@llnl.gov (M.A.M.); enright3@llnl.gov (H.A.E.); stewart66@llnl.gov (B.J.S.); loots1@llnl.gov (G.G.L.); 2 Center for Accelerator Mass Spectrometry, Lawrence Livermore National Laboratory, Livermore, CA 94550, USA; buchholz2@llnl.gov (B.A.B.); ognibene1@llnl.gov (T.J.O.); mccartt1@llnl.gov (A.D.M.); bench1@llnl.gov (G.B.); 3 Department of Internal Medicine, Division of Hematology and Oncology and UC Davis Comprehensive Cancer Center, University of California Davis Medical School, Sacramento, CA 95817, USA; mzimmermann@ucdavis.edu (M.Z.); tscharadin@ucdavis.edu (T.M.S.); bajonas@ucdavis.edu (B.A.J.); cxpan@ucdavis.edu (C.-X.P.); 4 Accelerated Medical Diagnostics Incorporated, Berkeley, CA 94708, USA; george_cimino@comcast.net

**Keywords:** accelerator mass spectrometry, cavity ring down spectrophotometry, radiocarbon, naphthalene, benzo[a]pyrene, cell turnover, triclocarban, metastasis, DNA adducts, biomarkers

## Abstract

This review summarizes recent developments in radiocarbon tracer technology and applications. Technologies covered include accelerator mass spectrometry (AMS), including conversion of samples to graphite, and rapid combustion to carbon dioxide to enable direct liquid sample analysis, coupling to HPLC for real-time AMS analysis, and combined molecular mass spectrometry and AMS for analyte identification and quantitation. Laser-based alternatives, such as cavity ring down spectrometry, are emerging to enable lower cost, higher throughput measurements of biological samples. Applications covered include radiocarbon dating, use of environmental atomic bomb pulse radiocarbon content for cell and protein age determination and turnover studies, and carbon source identification. Low dose toxicology applications reviewed include studies of naphthalene-DNA adduct formation, benzo[a]pyrene pharmacokinetics in humans, and triclocarban exposure and risk assessment. Cancer-related studies covered include the use of radiocarbon-labeled cells for better defining mechanisms of metastasis and the use of drug-DNA adducts as predictive biomarkers of response to chemotherapy.

## 1. Introduction

Radioisotopes play an important role in advancing our knowledge in the biomedical sciences. The applications are broad, ranging from positron-emission-tomography to the use of scintillation radiometry for determining protein turnover rates [1,2,3,4]. Nearly all radioisotope technologies detect and quantify radioisotopes based on the detection of a nuclear decay event. However, for many radioisotopes, this is an inefficient process, resulting in the need for high levels of radioactivity that are costly and require extensive safety precautions. For this reason, many investigators have avoided using radioisotopes in their research [5]. 

Radiocarbon-labeled drugs are often used to study absorption, distribution, metabolism, and excretion (ADME) in animals and humans. The resulting data aids in defining the metabolic fate of the drug and informs the usefulness of comparing pharmacokinetics (PK) and metabolism in animal models to humans. Radiocarbon (^14^C) is often the label of choice because it is stable enough to be incorporated into virtually any carbon position on a given molecule using standard organic chemical synthesis methodology, yet it is active enough of a β-emitter to be detected by standard techniques, such as liquid scintillation counting (LSC) [6]. The use of ^4^C has limitations due to the long half-life of this isotope (5740 years), which renders LCS counting of less than 5 to 10 picomoles ^4^C/mL of plasma or urine impractical [2], which renders the assessment of drugs at microgram doses impossible (for highly potent drugs or microdose studies).

Accelerator mass spectrometry (AMS) is a highly sensitive technology for quantifying radioisotopes that has more recently been applied to the biomedical sciences. Quantification of radioisotopes with AMS does not rely on the nuclear decay, but rather on the direct quantification of the isotopic nuclei through mass spectrometry (reviewed in [7]). This provides much greater sensitivity for isotope detection (10^3^ to 10^9^ times greater than decay counting), leading to the use of lower chemical and radioisotope doses and the analysis of smaller samples, which enables studies to be performed safely in humans, using exposures that are environmentally or therapeutically relevant while generating little radioactive waste. Most biomedical AMS studies have employed radiocarbon (^14^C) as the radiolabel, although the capability exists for detecting other isotopes, including ^3^H, ^26^Al, ^41^Ca, ^10^Be ^36^Cl, ^59^Ni, ^63^Ni, and ^129^I. Both ^14^C and ^3^H are commonly used in tracing studies because they can be readily incorporated into organic molecules, either synthetically or biosynthetically. This review will focus on the direct detection of ^14^C from biological samples, either using AMS or laser-based systems that do not rely on decay counting. Technologies that are reviewed herein include the conversion of samples to graphite or carbon dioxide, followed by direct AMS analysis, combined AMS/ion trap mass spectrometry, and laser-based quantitation, along with examples of applications of these technologies to molecular toxicology, cell turnover, metastasis, and chemotherapy drug resistance. 

## 2. Technology

### 2.1. Graphite 

The most important considerations in preparing samples for radiocarbon analysis are the amount of radioisotope in each sample, preventing contamination, and knowing the sources and amounts of any carbon introduced during processing. Numerous precautions need to be in place throughout the procedure to ensure the amount of isotope present is within the dynamic range of the spectrometer and to minimize the potential for contamination to ensure that the isotope detected in the sample is associated with the labeled compound under investigation. The sample of interest, for example, blood, DNA, or high-performance liquid chromatography (HPLC) fractions, must be converted to a form that is compatible with the ion source of the instrument. The standard procedure for the preparation of ^14^C-labled biological samples for AMS analysis is the conversion of the biological sample to graphite. This homogeneous state ensures uniformity and comparability between samples and standards that are compatible with the AMS ion source [8]. Graphite samples for AMS quantification are most commonly prepared by the reduction of carbon dioxide by hydrogen onto a catalytic iron or cobalt surface at temperatures around 500 °C [9,10,11,12,13]. Reduction of CO_2_ to a filamentous graphite using septa sealed vials proceeds rapidly and yields of >95% are routinely obtained. Samples containing as little as 20 µg of carbon can be converted to graphite [14]. The graphite produces intense, long-lasting negative ion beams upon introduction to the cesium sputter ion source with extremely small isotopic and mass fractionation. The graphite-preparation stage is the rate-limiting step in AMS studies and precludes real-time analyses, such as those possible with typical LC/MS methods. However, the recent development of a gas-accepting ion source has eliminated the need for graphite in some applications.

### 2.2. Gas Ionization

The elimination of matrix effects in the ^14^C-AMS analysis of biochemicals requires the physical and chemical equivalence for all measured carbon atoms. Additionally, almost all AMS systems use a cesium sputter ion source to generate negative ions and, consequently, methods for preparing biochemical samples for analysis were adapted from well-developed techniques used for geochronology studies. Subsequently, biochemical samples for AMS analysis are first combusted to CO_2_, followed by reduction to graphite. While these methods have proven to be very successful, the technique suffers from low throughput and requires significant and adroit human handling. The routine preparation of graphitic biochemical samples requires at least 0.5 mg of carbon and limits sensitivity to ~2 amol ^14^C/mg C (~0.3 mdpm/mg C). This requires that the analysis of biochemical mixtures separated using U/HPLC must be collected as discrete fractions with each treated as an individual sample. Samples from a single 30-minute LC trace can require several days to prepare and take over eight hours of AMS analysis time, costing several thousands of dollars, all of which increases if a higher resolution and/or duplicate analysis is required. In some instances, the number of samples from an LC trace can be reduced by collecting only fractions containing the peak(s) of interest and pooling fractions of “uninteresting regions”. However, this is not always a viable option, especially in instances where an entire metabolite profile is required.

Analysis systems that are compatible with the direct input of biochemical separation instrumentation, such as liquid chromatography, would allow real-time analysis, leading to increased resolution, minimal handling, and the ability to do molecule-specific tracing of small samples. This approach involves the direct introduction of carbon as CO_2_ into the ion source. This sample form is more efficient for the small samples common to biochemical research and allows for the direct interfacing of separation instrumentation to AMS. A moving wire interface, developed at Lawrence Livermore National Laboratory, provides one solution [15,16,17]. Briefly, the output of the HPLC is jetted onto a moving wire, which is pulled through a drying oven to remove the volatile solvent before introduction into a high temperature oven where the remaining analyte is combusted. The resultant CO_2_ gas is then carried in a helium stream to the ion source of the AMS spectrometer for ^14^C-quantification of the separated analyte.

**Figure 1:**
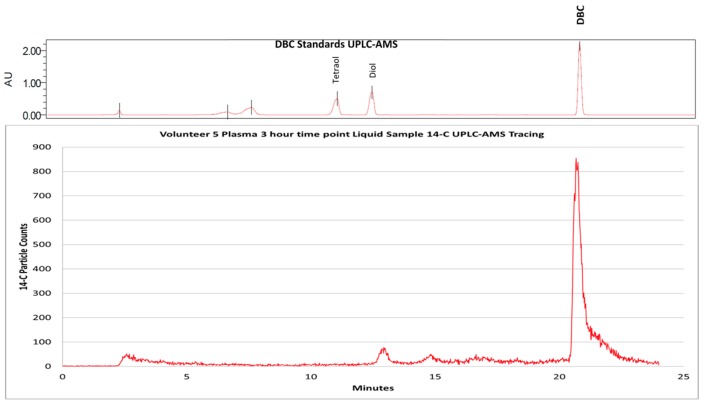
UHPLC-AMS of [^14^C]-DBC and putative metabolites from human plasma 3 h after and oral dose of 29 ng ^14^C-DBC. The use of unlabeled parent DBC and standards (DBC-(±)-11,12-diol and DBC-(±)-11,12,13,14-tetraol) (top graph) allow for the identification of ^14^C peaks in plasma (bottom graph).

The moving wire interface was used to measure human plasma and urine metabolism profiles following environmentally relevant exposures to the polycyclic aromatic hydrocarbons, dibenzo[def,p]chrysene (DBC) (Figure 1) [18] and benzo[a]pyrene [19]. Due to their toxicity, doses to healthy human volunteers were required to be kept as low as possible to minimize risk and the metabolite levels recorded following HPLC separation were so low that they could only have been measured as a CO_2_ gas. 

Aside from the direct coupling of HPLC-AMS, CO_2_ gas-capable ion sources have also been coupled with commercially available combustion furnaces for higher throughput analysis of discrete tracer biochemical samples. In the system described by van Duijn et al., discrete samples containing at least 70 µg carbon and at least 0.52 amol of ^14^C are combusted using an elemental analyzer, with the resultant CO_2_ captured and transferred to a gas-tight syringe for subsequent metering into a gas-accepting hybrid ion source on a 1 MV HVEE AMS spectrometer [20]. Up to 200 samples may be measured automatically in sequence, limited by the capacity of the sample target wheel of the ion source.

### 2.3. Parallel Accelerator and Molecular Mass Spectrometry (PAMMS)

**Figure 2:**
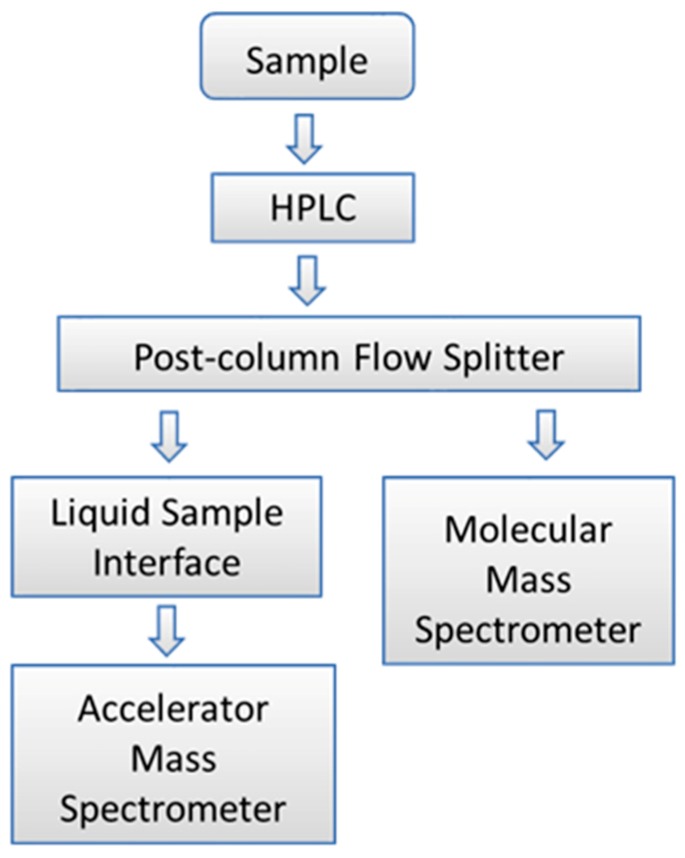
Block diagram showing the PAMMS instrument configuration.

Historically, AMS measurements have required the use of off-line orthogonal analytical techniques to speciate analytes measured by AMS. This limitation was based on the need to convert analytical samples to graphite prior to AMS measurement, destroying all chemical information in the process [13]. The recent development of the AMS liquid sample interface has enabled measurement of liquid samples without the need for graphitization [15,16,21]. The ability to measure liquid samples by AMS has also made it possible to integrate quantitative analysis with AMS sample measurements. The historical requirement for off-line speciation has been overcome by the recent development of a novel analytical technology that couples AMS directly with accurate mass spectrometry to enable real-time analysis of samples separated by high-performance liquid chromatography (HPLC). Based on the naming convention proposed by Sacks et al., this combined analytical method is referred to as parallel accelerator and molecular mass spectrometry (PAMMS) [22]. The LLNL PAMMS instrumentation is composed of a Waters Acquity H Class HPLC system, a Waters Xevo G2-XS QTOF instrument, an adjustable post-column flow splitter, a custom-built readout device, and the LS-AMS interface as depicted in the block diagram in Figure 2.

**Figure 3:**
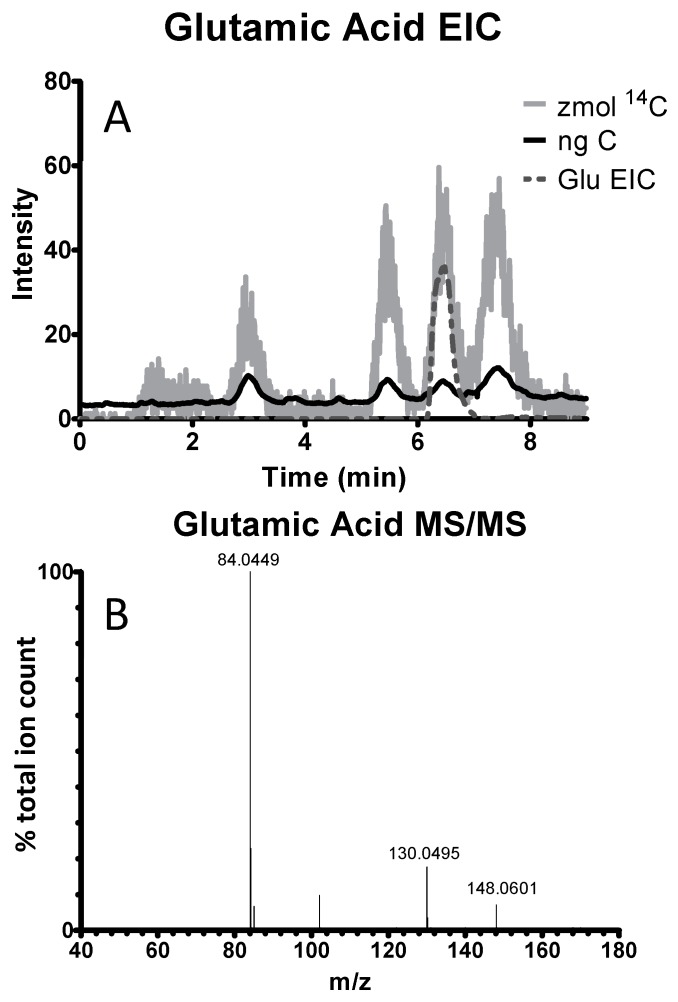
PAMMS analysis of carbon-14 labeled amino acid standards, showing (**A**) extracted ion chromatogram (EIC) and (**B**) mass spectrum for glutamic acid.

PAMMS provides accurate mass measurement and tandem mass spectrometry for structural elucidation of individual analytes separated by HPLC, but also measures stable carbon and carbon-14 in each separated analyte. PAMMS can therefore enable definitive identification, as well as quantitation, of each separated analyte. For example, Figure 3 shows separation of glutamic acid in a mixture of ^14^C-labeled amino acids, and identification based on the exact mass and MS/MS fragmentation pattern.

PAMMS represents a significant innovation that takes advantage of the ability of AMS to measure extremely low levels of ^14^C. The ability to use low concentrations of radiolabeled substrates in cells and organisms at concentrations accessible by AMS allows quantification of metabolites without perturbing normal metabolism and leads to more relevant quantification of metabolic rates and pathways. Coupling the sensitive isotope detection abilities of AMS with accurate mass spectrometry to identify analytes makes PAMMS a very powerful technique capable of providing both qualitative and quantitative metabolic measurements. Such measurements can improve risk assessment for toxicants and new therapeutic entities, deepen our understanding of xenobiotic and intermediary metabolism, help understand interactions between critical molecular pathways, and improve efforts to model and predict various metabolic and biological states when coupled with biocomputational methods. 

### 2.4. CRDS

AMS’s complexity, large size, time consuming sample processing, and relatively high cost have been an obstacle to the scientific community’s adoption of the method and its applications [23]. This has led to several scientific groups exploring new ways to measure ^14^C [24,25,26,27,28]. One of these methods, cavity ringdown spectroscopy (CRDS), has demonstrated ^14^C sensitivities below contemporary levels. Saturated-absorption cavity ring-down spectroscopy has achieved the greatest sensitivity of the CRDS techniques, with a minimum-detection limit 60-times smaller than the requirement for basic-biological studies [29]. 

Compared to AMS, CRDS is a simpler laser absorption technique for ^14^C detection, which leverages a high-finesse optical cavity constructed with high-reflectivity mirrors (<1 ppm losses). This setup permits gas–laser interaction path lengths equivalent to tens of kilometers and therefore, increased sensitivity. A measurement starts by coupling resonant laser light into the optical cavity. This light is then interrupted, and an exponential decay, or “ring-down”, is recorded on an optical detector. Differences between the characteristic decay time of empty and sample filled cavities are used to quantify the target species. 

For biological studies utilizing ^14^C, carbonaceous analytes are combusted into CO_2_ and introduced into the cavity. While CRDS does not have the sensitivity of AMS, several groups have demonstrated the sensitivity to resolve natural background ^14^C levels [29,30,31]. Furthermore, validation studies have been conducted, demonstrating CRDS produces results congruent with AMS when applied to duplicate samples [30,32]. 

## 3. Applications

### 3.1. Radiocarbon Dating

^14^C is produced naturally in the upper atmosphere by nuclear reactions between cosmic radiation and atmospheric gases, notably ^14^N. This natural ^14^C production rate varies slightly over time as the Earth’s magnetic field changes and the cosmic ray fluxes fluctuate, but it has remained relatively constant over most of recorded history, producing a natural source of ^14^CO_2_ that subsequently labels every living thing on Earth as carbon moves through the food chain and carbon cycle. As long as a plant or animal is alive, it is replenishing or increasing its carbon either directly from the atmosphere (plants) or indirectly through consumption of plants and other animals. When an organism dies, the carbon replacement ceases. Since ^14^C is radioactive (half-life T_1/2_ = 5730 y), the decrease in the ^14^C/C concentration in tissue or biological structures compared to the atmospheric record can be used to determine how long an organism has been dead. Willard Libby was awarded the 1960 Nobel Prize in Chemistry for the development of radiocarbon dating [33].

### 3.2. Bomb Pulse Dating

**Figure 4:**
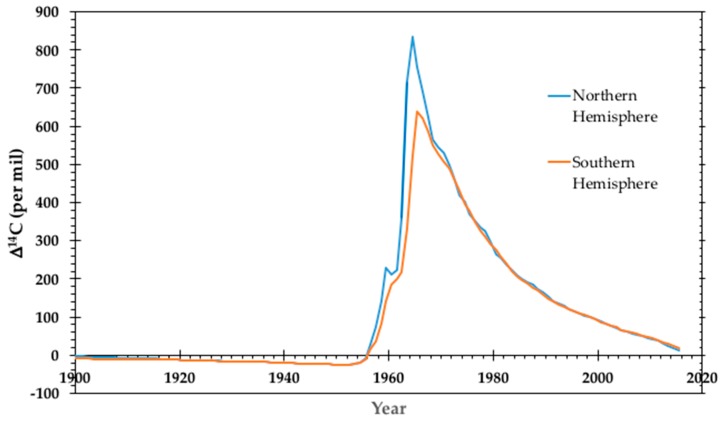
Annual averages of atmospheric ^14^C/C for the northern and southern hemispheres. Data before 1959 is derived from plant material while data from 1959 to present is derived from atmospheric CO_2_ collections and plant material. Data is reported in the Δ^14^C convention described by Stuiver and Polach [38].

Above-ground testing of nuclear weapons produced an anthropogenic spike in atmospheric ^14^CO_2_ and consequently produced a small, but measurable excess ^14^C label in every living thing on the planet. This spike in ^14^C is generally called the radiocarbon bomb pulse. The pulse nearly doubled the natural atmospheric ^14^C between 1955 and 1963, when the Limited Test Ban Treaty ended atmospheric, under water, and outer space detonations by the United States, Soviet Union, and the United Kingdom. Since the peak in 1963, atmospheric ^14^CO_2_ has been decreasing as carbon moves into the biosphere and marine reservoirs and the burning of ^14^C-free fossil fuels drives the atmosphere to pre-bomb ^14^C in 2018 to 2019. The date of biological molecule synthesis is highly correlated to atmospheric ^14^CO_2_, so the atmospheric record is a chronometer of the molecular age or carbon source. Figure 4 depicts the ratio of atmospheric radiocarbon to total carbon (^14^C/C) from 1900 to 2015 for the northern and southern hemispheres based on compiled data [34]. The ^14^C/C differs between the hemispheres since the weapons tests were conducted at relatively few locations, mostly in the northern hemisphere. Before 1955 and after 1970, there is little difference in the annual averages of the hemispheres.

#### 3.2.1. Carbon Source Determination

Since fossil-derived carbon is devoid of ^14^C, chemicals produced from petroleum do not contain ^14^C. Chemicals, vitamins, or food additives from a “natural” biological source possess the atmospheric ^14^CO_2_ signature. The ^14^C/C of an “all-natural” or “real” product should, therefore, be consistent with 100% biologically sourced material. If fossil-derived carbon is added to the product, the ^14^C/C is depressed and easily measured by AMS. For example, in a 2011 study, real vanilla extracted from vanilla beans possessed a contemporary radiocarbon signature of F^14^C = 1.059 ± 0.004 modern while imitation vanilla had a F^14^C = 0.038 ± 0.001 modern [35,36]. The analysis of ^14^C can determine if a food or personal care product has been adulterated with synthetic compounds. The technique can also be used to determine if packaging contaminates a food product with an unintended or undesirable compound. When a compound, such as phthalate, is found in a food product, ^14^C AMS analysis of the purified compound can determine if it is naturally occurring in the food product, a result of leaching from packaging, or a combination of these. Analyses of stilton cheese and butter for bis(2-ethylhexyl) phthalate (DEHP) found that about 24% and 16% of the DEHP in the dairy products were of biological origin and not from packaging materials [36,37].

#### 3.2.2. Structural and Pathological Protein Dating

The standard method to date bone found at archeological sites is to demineralize the bone, extract the collagen, and use an ultrafiltration procedure to exclude smaller fragments, typically under 30 kD. Collagen extraction works well because it is resistant to diagenesis (mineral exchange) of the mineral component of bone while being protected by the mineral structure. Collagen is not static in bone, it turns over as bone is remodeled throughout life, with the rates of turnover varying with age, type of bone, position within a bone, physical activity, etc. Hence, the ^14^C of bone collagen is an integration over a lifetime of variable inputs and outputs. The changing ^14^C/C in the bomb pulse enables studies to determine the approximate turnover in bone [39] and opens forensic applications for approximating the ages of skeletal remains [40].

Many structural proteins are found outside of bone. Many of these structural proteins are found in the extra-cellular matrix (ECM) of organs, muscle, cartilage, ligaments, and vasculature. Human lung parenchymal elastic fibers were shown to be the age of the person using ^14^C analyses and aspartate racemization [41]. Radiocarbon dating of collagen from Achilles tendon and human articular cartilage shows growth and turnover through adolescence, but virtually no turnover during adulthood [42,43]. The eye lens continues to grow throughout life adding new cells to the outside in series of layers. The crystallin proteins that provide much of the structure of the lens have been shown to have very little turnover throughout life [44,45].

The chronological deposition of pathological structures is approachable by bomb pulse dating. In the progression of Alzheimer’s Disease, the pathological structures of neurofibrillary tangles (NFT) and senile plaques (SP) accumulate over time. Post mortem analyses of the separated structures provided an average age, weighted by the rate of accumulation [46]. The average ages of the SP and NFT predated clinical symptoms of Alzheimer’s disease in half the cases, indicating significant accumulation before cognitive deterioration. In another example of long-term accumulation, arterial plaques that restrict blood flow have also been shown to develop over decades [47,48]. The collagen extracted from excised cerebral aneurysms tends to be about 3 years old, even for aneurysms followed by imaging for years [49,50]. Additionally, subjects with risk factors of smoking, cocaine use, and hypertension had collagen within a year old, indicating very rapid carbon turnover [49].

#### 3.2.3. Cell Lifetime and Turnover

Genomic DNA only acquires significant new carbon at cell division, so the ^14^C/C of nuclear DNA is a metric of the cell birth date [51]. Cell or nuclei surface markers can be utilized using fluorescence activated cell sorting to isolate specific cell types for analyses. DNA is isolated from specific cell populations, rinsed thoroughly to remove residual solvents, checked for purity using UV/Vis absorbance, processed for AMS analyses using high precision natural radiocarbon preparation techniques, and measured by AMS. Genomic DNA dating has been used to investigate neurogenesis throughout many regions of the human brain [51,52,53,54,55]. The lack of bomb pulse carbon in neuronal DNA of subjects born before 1955 indicated that DNA repair provides an insignificant amount of new carbon after cell division [51]. It has also been used to determine that adipocytes turnover approximately every 10 years [56] while the lipids they hold cycle every 1.5 years [57] and cardiomyocytes turnover at a low rate [58]. Using bromodeoxyuridine (BrdU) and ^14^C/C analyses of DNA from pancreatic β-cells, it was determined that insulin producing β-cells turnover at a 1% to 2% annual rate through early adulthood and then cease to turnover after the age of 30 [59]. Antibody-secreting plasma cells have been found to persist for decades in the intestines although antibodies last only several weeks in circulation [60]. An alternative to dating DNA, dating histones established histone turnover as a critical regulator of cell type-specific transcription and plasticity in the mammalian brain [61].

### 3.3. Tracking the Fate of Cells Labeled with [^14^C]Thymidine

**Figure 5:**
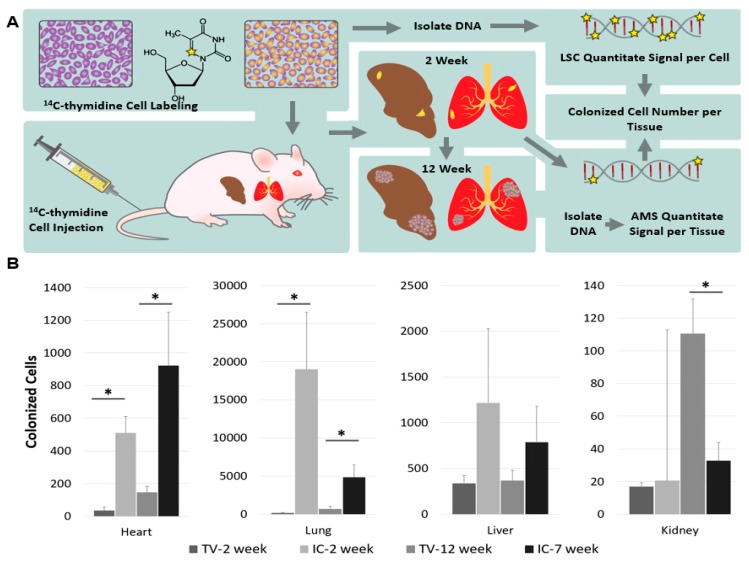
Workflow and validation of ^14^C-labeling cancer colonization assay. (**A**) Schematic of colonization assay workflow. Cells were first cultured with ^14^C-thymidine media to achieve single cell resolution and injected into NSG mice via the tail vein (TV), heart (IC), or subcutaneous (SQ) routes of delivery. Injected cells were allowed to metastasize for up to 12 weeks. Tissues were harvested at early (2 weeks post injection) and late (12 weeks post injection) time points and DNA was isolated and quantified using AMS. In parallel, the activity of ^14^C-thymidine label in cultured cells was quantified using liquid scintillation counting (LSC). AMS measurements and LSC readings were combined to calculate the number of colonized cells per each organ examined. (**B**) Tail vein and intracardiac injected cancer cell colonization. Profile of colonized cells in target tissues calculated from ^14^C signal in DNA from target tissues isolated at 2-weeks post injection, 7-weeks for intracardiac (IC), or 12-weeks for tail vein (TV) (*n* = 5).

Using a similar principle, we can also quantify the number of cancer cells that colonize distant sites to form metastatic tumors [62]. In a recent publication, we have taken cancer cell lines with varying metastatic potential, and labeled them in vitro with ^14^C-thymidine, such that we could identify by AMS a single labeled cell among 1 million unlabeled cells. These labeled cells were introduced in mice via various routes known to produce metastatic tumors (tail vein, TV; intracardiac, IC) and after 2-weeks or 12-weeks post injection, all organs were examined for the presence of metastatic tumors. Whether visible tumors were present or not, total DNA was isolated from each organ, and the total carbon was examined for the presence of ^14^C. The amounts of ^14^C detected per organ were referred back to the amount of ^14^C present per cell, prior to injection into the mice, to determine how many cells traveled to distant sites and initiated a metastatic tumor (Figure 5A). 

Using this approach, we determined that less than 5% of human cancer cells injected into immunodeficient mice form subcutaneous tumors, and even fewer cells initiate metastatic tumors. Comparisons of metastatic site colonization between a highly metastatic (PC3) and a non-metastatic (LnCap) prostate cancer cell line showed that PC3 cells colonize target tissues in greater quantities at 2 weeks post-delivery, and by 12 weeks post-delivery, no ^14^C was detected in LnCap xenografts, suggesting that all metastatic cells were cleared (Figure 5B). The ^14^C-signal correlated with the presence and the severity of metastatic tumors. AMS measurements of ^14^C-labeled cells provides a highly-sensitive, quantitative assay to experimentally evaluate metastasis and colonization of target tissues in xenograft mouse models. In the future, this approach could potentially be adapted to evaluate tumor aggressiveness and assist in making informed decisions regarding treatment, towards a more informed personalized therapy regimen.

### 3.4. Low Dose Toxicity

One of the biggest advantages of AMS is the ability to perform low dose toxicity studies, which allow for the assessment of chemicals at environmentally relevant dose levels. Numerous studies have used AMS to investigate the biodisposition of chemicals at low-dose human exposure levels. Below are examples of some of these studies. 

#### 3.4.1. Naphthalene

Naphthalene (NA) is ubiquitous in both the indoor and outdoor environment. Common sources of NA exposure include combustion products from vehicle emissions, biomass, cigarettes and wildfires, mothballs, and house-hold block deodorizers. Naphthalene metabolites have been detected in the urine of nearly all children and adults tested, regardless of locale or occupation [63]. Further, studies in children have shown increased chromosomal aberrations that correlate with urinary markers for NA exposure, but these studies cannot establish a cause and effect relationship [63]. Furthermore, NA exposure caused bronchiolar alveolar carcinomas in female mice and neuroblastomas in the nasal epithelium of rats in the National Toxicology Program carcinogenesis bioassays [64,65]. The mechanism of cancer initiation is unclear, however, so investigations of protein adducts, DNA adducts, and repair tolerance [66,67] have been conducted using NA and its metabolite, 1,2 naphthoquinone (NQ). Using well established techniques to obtain metabolically active, live tissue samples [68], freshly micro-dissected respiratory tissues were incubated with NA or NQ at 250 μM, calculated as equivalent to the tissue concentration obtained from exposure to the 10 ppm OSHA exposure limit for NA [69]. DNA adducts of NA and NQ are present in low levels, but protein adducts are much more common [66,67]. The technique of ex vivo exposure of metabolically active tissue avoided making an aerosol of a ^14^C-lableled toxic chemical and is applicable to other inhalation hazards.

#### 3.4.2. Triclocarban

In a recently published study by Enright et al., the potential of an environmentally relevant concentration of the antimicrobial, triclocarban (TCC), to transfer from the mother to the offspring during development was evaluated using AMS [70]. Triclocarban is an antimicrobial found in many personal care products (i.e., deodorants, soaps) and is among the top 10 most commonly detected wastewater contaminants [71,72]. Given its prevalence in the environment, bioaccumulation of TCC has been observed and reproductive effects have been noted as a result from exposure [73]. Exposure to compounds, such as TCC, during development may have deleterious consequences to the developing embryo and fetus, given their heightened sensitivity to perturbations in hormone levels and immature protective mechanisms (i.e., liver metabolism, DNA repair mechanisms). 

In this study, ^14^C-labeled TCC (100 nM) was administered to CD-1 mouse dams through their drinking water up to gestation day 18, or from birth through to postnatal day (PND) 10. 

**Figure 6:**
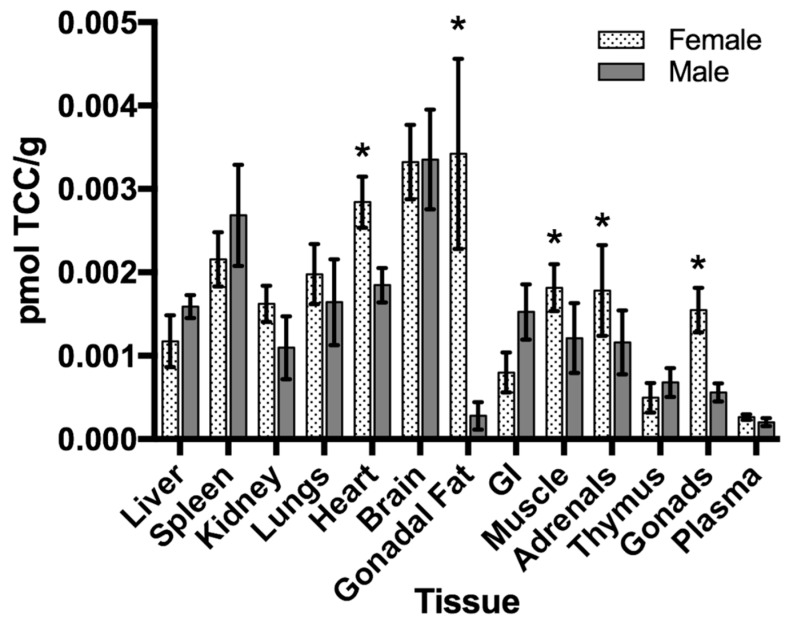
Tissue distribution of 14C-TCC in exposed offspring at postnatal day 42. Data is expressed as pmol of TCC/gram of tissue ± SEM (*n* = 5/sex). * *p* < 0.05, when comparing female to male offspring.

Using AMS, the concentration of TCC was determined in both offspring and dams after exposure; TCC transferred from mother to offspring both trans-placentally (0.005% ± 0.001% ingested dose/gram (%ID/g) and through lactation (0.015% ID/g ± 0.002%) (Figure 6). The three-fold higher concentration in offspring after exposure through lactation (*p* = 0.003) demonstrated that TCC readily transfers through breast milk. After exposure through lactation, TCC exposed offspring were heavier in weight than unexposed controls (*p* = 0.016 for PND21–56), with females more affected (11% increase) than males (8.5% increase) (data not shown). Tissue accumulation was also quantified using AMS at 6 weeks post exposure. TCC-related compounds were detected in tissues with higher concentrations observed in the brain, heart, and fat. Quantitative real-time polymerase chain reaction (qPCR) of liver and fat tissue suggested alterations of lipid metabolism in exposed female offspring; this was further supported by an increase in fat pad weights and hepatic triglycerides. This was the first report quantifying the translocation of an environmentally relevant concentration of TCC from mother to offspring; this study was enabled by the high sensitivity of AMS. Taken together, our findings suggest that TCC readily transfers from the mother to the offspring and that early-life exposure may interfere with lipid metabolism, which can ultimately have implications for human health. 

#### 3.4.3. Benzo[a]pyrene

Benzo[a]pyrene is a widely studied polycyclic aromatic hydrocarbon (PAH) that has been shown to induce cardiovascular, developmental, immunological, and reproductive disorders in model systems [74,75,76]. It has also been implicated as a human carcinogen and is an environmental chemical of concern for human exposure according to the Agency for Toxic Substances and Disease Registry [77]. Using the recently developed on-line UPLC-AMS interface with the gas accepting ion source, human pharmacokinetic and metabolite profiles were determined from microdose exposures of BaP. Five human volunteers were exposed to an oral dose of 46 ng (5 nCi) of ^14^C-BaP. Blood was collected at given time intervals and pharmacokinetic and metabolite parameters were quantified by AMS. At the dose used, BaP was fairly rapidly eliminated from the plasma and very little parent compound was present in the plasma even at the earliest time point examined, indicating extensive metabolism of BaP in these human subjects. The use of the UPLC-AMS together with the on-line gas accepting ion source provided exquisite sensitivity (zepto-mole^14^C in biological samples), allowing for the quantification of BaP plasma metabolites of BaP from exposure levels that were 5 to 15 times lower than the estimated daily exposure to BaP [19]. 

### 3.5. Diagnostic Microdosing: Using Drug-DNA Adducts as Biomarkers of Chemotherapy Response

Chemotherapy drugs that modify DNA are a cornerstone of modern cancer treatment and are used in nearly half of all cancer patients [78,79]. However, their efficacy is limited by severe side effects and intrinsic or acquired drug resistance, eventually causing treatment failure [78,80,81,82]. AMS has been used over the past 15 years for the measurement of drug–DNA interactions, and the resulting data have been correlated with cell sensitivity and/or tumor response in mice and humans [83,84,85,86,87,88,89,90,91,92,93,94,95,96]. The overarching hypotheses of this work are that a threshold level of drug–DNA adducts are required for cell killing and clinical response, and that microdose induced drug–DNA adduct levels are predictive of the cellular capacity to achieve such a threshold upon therapeutic dosing. This approach, known as “diagnostic microdosing”, has been initially demonstrated for platinum-based chemotherapy for the treatment of solid tumors and induction chemotherapy for leukemia. Based on this and other work, it is clear that for some chemotherapeutics, microdose-induced drug-DNA adducts are predictive of drug sensitivity and response in cell culture and mouse tumor xenograft studies, along with an extension of this effort to two pilot clinical studies focused on platinum-based chemotherapy (clinicaltrials.gov identifier NCT01261299 and NCT02569723) and a retrospective study on viably cryopreserved human acute myeloid leukemia (AML) cells. 

The diagnostic test protocol consists of four steps: (1) Creation of the individualized biomarkers in patient cells by exposure to ^14^C-radiolabeled drugs, (2) isolation of DNA containing the biomarkers, (3) determination of the ^14^C associated with the DNA via AMS analysis, and (4) comparison of the patient’s drug-DNA adduct levels to a database of clinical responses in order to assign a predictive score that indicates the probability of response.

Three key observations have included: (a) The level of drug-DNA adducts is generally low in resistant cancers and high in responsive cancers, (b) microdosing predicts the level of therapeutic-induced drug-DNA adducts, and (c) microdose-induced DNA adduct frequencies correlate with cellular drug sensitivity and patient response. Selected published examples of these results are summarized below. 

#### 3.5.1. Predicting Response to Platinum-Based Therapy with Microdosing

**Figure 7:**
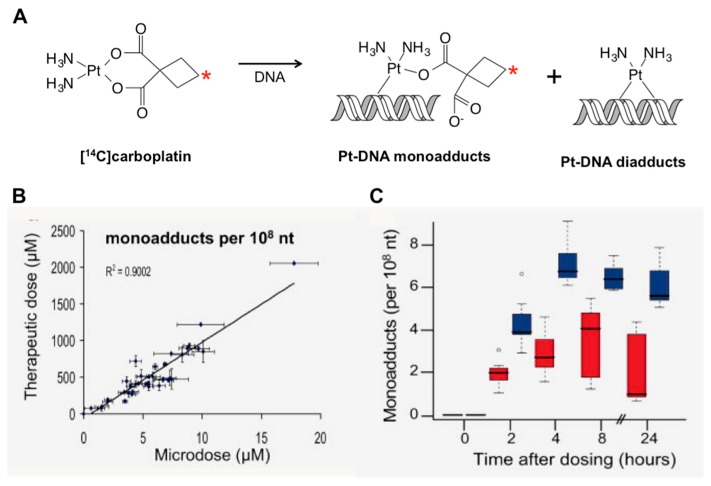
Correlation of microdose-induced [^14^C]carboplatin-DNA adduct levels to therapeutic dose-induced adduct levels in cancer cell lines. (**A**) Diagram of carboplatin-DNA adduct formation. (**B**) Linear regression of microdose-induced versus therapeutic dose-induced carboplatin-DNA adducts. (**C**) Sensitive cell lines (blue) have significantly higher carboplatin-DNA adduct levels than resistant cancer cell lines (red).

Figure 7 shows cell culture data for a set of six cancer cell lines dosed with [^14^C]carboplatin. The drug interacts with DNA to form [^14^C]carboplatin-DNA “monoadducts” that are measurable by AMS (Figure 7A). The monoadduct levels formed by microdoses were linearly proportional to those formed by therapeutically relevant concentrations of the drug in the media over 24 hours (Figure 7B). This is an important observation, since it implies that monoadduct levels formed from microdoses are likely to be predictive of those induced by therapeutic doses in patients. Half of the cell lines tested had carboplatin IC_50_ values below 100 μM (approximately the in vivo C_max_ in humans) and were assigned as “sensitive”. The remaining cell lines were designated as “resistant”. The sensitive and resistant cell lines could be significantly differentiated based on microdose-induced carboplatin monoadduct levels (Figure 7C), establishing proof of concept and justifying in vivo studies in mice and humans.

**Figure 8:**
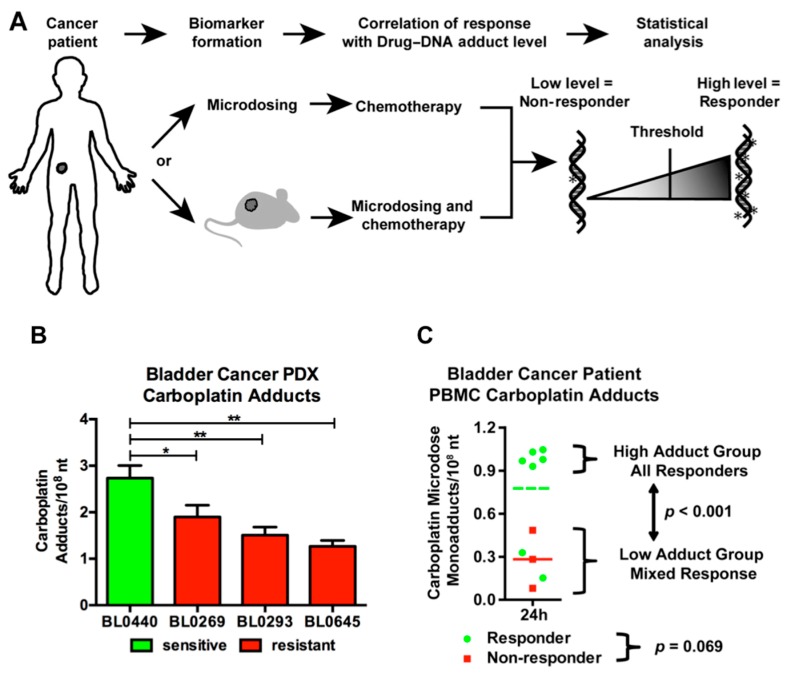
Correlation of microdose-induced [^14^C]carboplatin-DNA adduct levels to therapy response in bladder cancer PDX and patients. (**A**) Clinical study design. (**B**) Carboplatin-DNA adduct levels are significantly higher in the sensitive bladder cancer PDX model (green) than in the resistant models (red). (**C**) Correlation of PBMC [^14^C]carboplatin-DNA adduct levels to the response in 10 bladder cancer patients (green = responder, red = non-responders, line = mean adduct level).

Figure 8 shows data supporting the correlation between drug-DNA adduct levels and in vivo treatment response in mice and humans. Mice bearing patient derived bladder tumor xenografts (PDX) were established as depicted in Figure 8A. There was a significant correlation between microdose-induced carboplatin-DNA monoadduct levels and tumor growth inhibition of platinum-based chemotherapy (Figure 8B). The pilot clinical trial accrued 10 bladder cancer patients (stage II and higher), for whom platinum-based chemotherapy was administered. Patients were administered approximately 1% of the therapeutic dose of [^14^C]carboplatin (a microdose), followed by blood sampling within 24 h. DNA isolated from peripheral blood mononuclear cells (PBMC) was assessed for carboplatin-DNA adduct levels by AMS. Within three months of the microdosing assay, patients began standard of care chemotherapy regimens consisting of either cisplatin or carboplatin in combination with other drugs (typically gemcitabine (GC) or methotrexate, vinblastine, and doxorubicin (MVAC)). Patient response was determined at the time of cystectomy—typically after three cycles of chemotherapy. A patient whose tumor burden in the bladder was reduced to pT1 or less was considered a responder and a patient with a pT2 or greater tumor was considered a non-responder, based on standard RECIST criteria [97]. The main endpoint of the study was to demonstrate a significant difference in the mean drug-DNA adduct levels in PBMC (a surrogate for tumor tissue) between responders and non-responders. Seven patients responded to chemotherapy (green circles), whereas three patients showed disease progression (red squares) (Figure 8C). Responders exhibited approximately 2-fold higher mean monoadduct levels (green line) than non-responders (red line) (0.741 ± 0.346 vs. 0.283 ± 0.202 monoadducts/10^8^ nt, respectively, *p* = 0.069). The drug-DNA adducts were distributed in two distinct groups; high (0.941 ± 0.030 adducts per 10^8^ nt) and low (0.266 ± 0.158 adducts per 10^8^ nt) drug-DNA adduct levels with a statistically significant difference (*p* < 0.001). All five patients in the high adduct level group responded to chemotherapy, which is a 100% positive predictive value (PPV) for this group. The low adduct level group included three non-responders and two responders. The results of this clinical trial show a clear trend for responders to have higher Pt-DNA adduct levels 24 h after microdose administration, which supports the feasibility of patient stratification by the diagnostic microdosing approach.

#### 3.5.2. Ex Vivo Diagnostic Microdosing for Predicting Response to 7 + 3 in AML Patients

In contrast to our previous efforts that focused on administering microdoses to patients, our more recent work has focused on establishing proof-of-concept for a lab-based test in which biobanked or fresh patient leukemia patient samples are dosed ex vivo. This change will allow us to overcome our past difficulty in accruing patients that were unwilling to undergo IV administration of a radiolabeled drug that would not provide a direct benefit (a non-interventional study). Furthermore, ex vivo microdosing allows us to analyze multiple drug regimens on each patient sample. AML is ideal for this concept, since it is a “liquid tumor” that can easily be accessed via a blood draw or bone marrow biopsy, and is predominantly treated with two drugs that both interact with DNA. 

**Figure 9:**
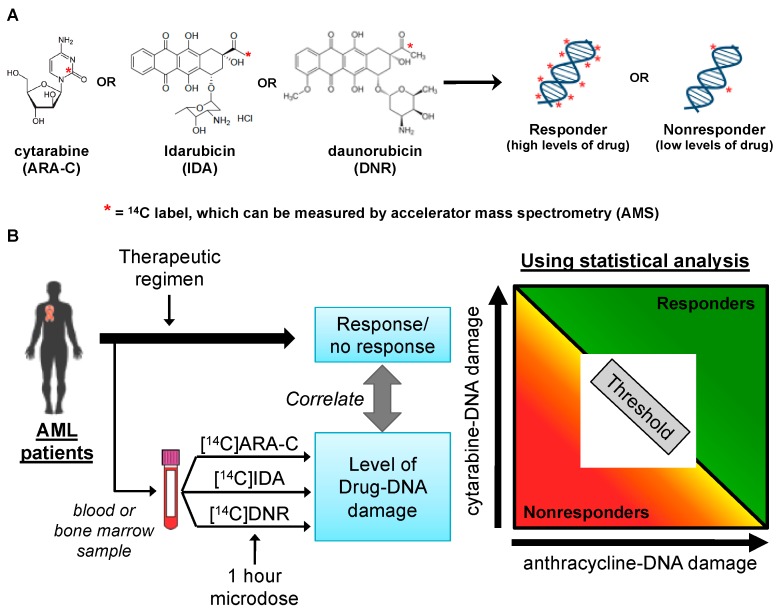
Overview of the ex vivo “diagnostic microdosing” strategy. (**A**) Radiocarbon-labeled cytarabine (ARA-C), idarubicin (IDA), or daunorubicin (DNR) bind to or are incorporated into AML DNA in proportion to the cellular sensitivity to each drug. The resulting drug-DNA “adducts” can be quantified by accelerator mass spectrometry (AMS). (**B**) Strategy for using in vitro microdosing to predict AML patient response to 7 + 3 chemotherapy. Cells isolated from a blood draw or bone marrow (fresh or viably cryopreserved) are briefly exposed to microdoses of each drug (triplicate wells per drug) and assessed by mass spectrometry for quantitation of drug-DNA adduct levels as biomarkers of clinical response to 7 + 3 induction chemotherapy.

The most effective therapy for AML is treatment with “induction chemotherapy” with two DNA damaging drugs, including the antimetabolite, cytarabine (ARA-C), and an anthracycline, such as daunorubicin (DNR) or idarubicin (IDA)—structures shown in Figure 9A. This regimen is known as 7 + 3 (7 days of continuous infusion ARA-C and 3 days of bolus DNR or IDA), and is the standard of care for up to two thirds of AML patients. Treatment is started as soon as possible, typically within 5 to 7 days of diagnosis [98,99,100,101]. In addition, a subset of patients, including eligible younger patients and relapsed or refractory (R/R) patients, can be treated with a combination of high-dose bolus ARA-C and DNR or IDA, known as 3 + 4 [102,103,104,105]. Patients who are not eligible for 7 + 3 are typically placed on a less toxic, ARA-C-containing regimen.

Basic research into the significance of drug–DNA adducts in patients treated with anthracycline derivatives and antimetabolites similar to IDA [106] and ARA-C [107] chemotherapies has been reported, but none of these findings have been translated into clinical use [108,109,110,111,112,113]. Several reports have documented associations between drug–DNA adduct levels and clinical response and overall survival [93,114]. These reports support the concept that a predictive stratification strategy can be used to personalize chemotherapy if the capacity for cells to form high levels of drug-DNA adducts can be predicted prior to the initiation of therapy. This body of work is being adapted to predicting AML patient responses to 7 + 3 induction chemotherapy by implementing a diagnostic microdosing test (Figure 9B). 

**Figure 10:**
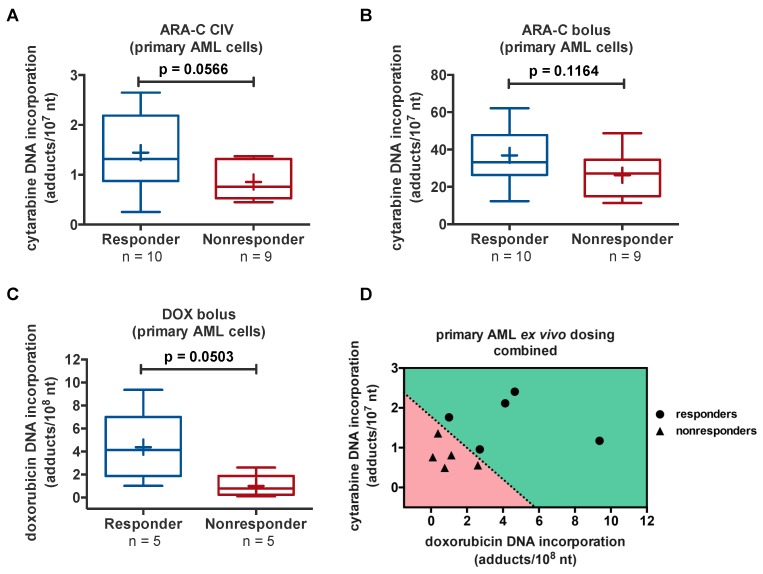
Correlation of ARA-C- and DOX-DNA levels to 7 + 3 response after in vitro dosing of 20 primary AML samples. PBMC were exposed to either exposed to a microdose of [^14^C]ARA-C or [^14^C]DOX at ~1% of the approximate plasma C_max_ obtained with ARA-C CIV (**A**), ARA-C bolus (**B**), or DOX bolus (**C**) observed in patients. Cells were dosed for 1 h followed by DNA isolation and AMS analysis. These data show proof of principle that diagnostic microdosing is useful for predicting patient response to 7 + 3 chemotherapy, but a larger confirmatory study is necessary. Furthermore, the ARA-C and DOX-DNA adducts can be plotted together to differentiate responders and nonresponders—average adduct levels for each patient are shown for simplicity (**D**).

After optimization of the protocols using cell culture experiments, we performed the diagnostic microdosing protocol on 19 clinically annotated viably cryopreserved primary human AML samples, including 10 responders and nine nonresponders to 7 + 3 induction chemotherapy, and DOX protocol on 10 primary AML samples. When the primary samples were grouped based on patient response, the responsive patients had higher mean drug–DNA adduct levels compared to the nonresponders for all dosing regimens (Figure 10A–C). Statistical differences between the responders and nonresponders were determined by unpaired *t*-tests with *p* < 0.05 as the statistically significant cutoff. The ARA-C- and DOX-DNA adduct levels from each primary AML sample when plotted together showed a complete separation in responders and nonresponders (Figure 10D—for 10 patients, since DOX- and ARA-C combined adduct data are currently only available for 10 patients).

In summary, the ability to quantitate the DNA incorporation of ARA-C and DOX in paired sensitive and resistant cell lines was demonstrated, and higher drug incorporation rates in the more sensitive cell lines was observed. Furthermore, similar correlations were observed in AML cell lines and with patient response primary AML samples. These preliminary data show that the adduct levels indeed correlate with resistance to doxorubicin and ARA-C. 

## 4. Conclusions

Although much of the scientific demonstrations of the technological advances reviewed herein for radiocarbon tracing are outside the scope of drug development and toxicology, there are clear implications for such studies. Drug discovery, early clinical development, and studies to support regulatory approval can all benefit from higher sensitivity and low-cost, high throughput radiotracer technologies [115,116,117,118]. For example, in human studies, with much lower radiocarbon doses that are currently used for HPLC-LSC, studies are not only technically desirable, but are also more ethical since healthy human volunteers would have reduced radiation exposures. Absolute bioavailability for oral compounds is another obvious application that would benefit from improved radiotracing technologies. Although “standard” LC-MS is sufficient for PK and ADME studies of most drug candidates, there will always be a subset of efficacious lead compounds that possess very high potency at very low doses or with unusual PK or biodistribution that require radiolabel studies. Therefore, drug development is likely to be more successful as radiotracer technologies become more accessible and accepted. Radiocarbon tracer technology continues to evolve in order to increase throughput, reduce cost and the amount of radioactivity needed, and broaden the types of applications that can be accommodated. Basic science, environmental and molecular toxicology, and clinical translational applications continue to be uniquely enabled by radiotracer studies, which are critical for advancing our understanding of fundamental biology, protecting the environment, and improving human health. 

## References

[1] Abramson F.P. (1994). Crims—Chemical-Reaction Interface Mass-Spectrometry. Mass Spectrom. Rev..

[2] Rosler H. (1995). The Impact of W. K. Rontgen’s Discovery on the Use of Internalizable Sources of Ionizing Energy in Diagnostic and Therapeutic Nuclear-Medicine. Experientia.

[3] Devries R.A., Debruin M., Marx J.J.M., Vandewiel A. (1993). Radioisotopic Labels for Blood-Cell Survival Studies—A Review. Nucl. Med. Biol..

[4] Young V.R., Ajami A. (1999). Isotopes in nutrition research. Proc. Nutr. Soc..

[5] Mayer A., Neuenhofer S. (1994). Luminescent Labels—More Than Just an Alternative to Radioisotopes. Angew. Chem. Int. Ed..

[6] Garner R.C., Barker J., Flavell C., Garner J.V., Whattam M., Young G.C., Cussans N., Jezequel S., Leong D. (2000). A validation study comparing accelerator MS and liquid scintillation counting for analysis of 14C-labelled drugs in plasma, urine and faecal extracts. J. Pharm. Biomed. Anal..

[7] Turteltaub K.W., Vogel J.S. (2000). Bioanalytical applications of accelerator mass spectrometry for pharmaceutical research. Curr. Pharm. Des..

[8] Vogel J.S., Turteltaub K.W., Finkel R., Nelson D.E. (1995). Accelerator mass spectrometry. Anal. Chem..

[9] Vogel J.S., Southon J.R., Nelson D.E., Brown T.A. (1984). Performance of Catalytically Condensed Carbon for Use in Accelerator Mass-Spectrometry. Nucl. Instrum. Methods B.

[10] Vogel J.S., Nelson D.E., Southon J.R. (1987). C-14 Background Levels in an Accelerator Mass-Spectrometry System. Radiocarbon.

[11] Vogel J.S., Southon J.R., Nelson D.E. (1987). Catalyst and Binder Effects in the Use of Filamentous Graphite for Ams. Nucl. Instrum. Methods B.

[12] Vogel J.S. (1992). Rapid Production of Graphite without Contamination for Biomedical Ams. Radiocarbon.

[13] Wilson A.T. (1992). A Simple Technique for Converting CO_2_ to AMS Target Graphite. Radiocarbon.

[14] Ognibene T.J., Bench G., Vogel J.S., Peaslee G.F., Murov S. (2003). A high-throughput method for the conversion of CO^2^ obtained from biochemical samples to graphite in septa-sealed vials for quantification of ^14^C via accelerator mass spectrometry. Anal. Chem..

[15] Thomas A.T., Ognibene T., Daley P., Turteltaub K., Radousky H., Bench G. (2011). Ultrahigh efficiency moving wire combustion interface for online coupling of high-performance liquid chromatography (HPLC). Anal. Chem..

[16] Thomas A.T., Stewart B.J., Ognibene T.J., Turteltaub K.W., Bench G. (2013). Directly coupled high-performance liquid chromatography-accelerator mass spectrometry measurement of chemically modified protein and peptides. Anal. Chem..

[17] Ognibene T.J., Thomas A.T., Daley P.F., Bench G., Turteltaub K.W. (2015). An Interface for the Direct Coupling of Small Liquid Samples to AMS. Nucl. Instrum. Methods Phys. Res. B.

[18] Madeen E.P., Ognibene T.J., Corley R.A., McQuistan T.J., Henderson M.C., Baird W.M., Bench G., Turteltaub K.W., Williams D.E. (2016). Human Microdosing with Carcinogenic Polycyclic Aromatic Hydrocarbons: In Vivo Pharmacokinetics of Dibenzo[def,p]chrysene and Metabolites by UPLC Accelerator Mass Spectrometry. Chem. Res. Toxicol..

[19] Madeen E., Siddens L.K., Uesugi S., McQuistan T., Corley R.A., Smith J., Waters K.M., Tilton S.C., Anderson K.A., Ognibene T. (2019). Toxicokinetics of benzo [a] pyrene in humans: Extensive metabolism as determined by UPLC-accelerator mass spectrometry following oral micro-dosing. Toxicol. Appl. Pharm..

[20] van Duijn E., Sandman H., Grossouw D., Mocking J.A.J., Coulier L., Vaes W.H.J. (2014). Automated Combustion Accelerator Mass Spectrometry for the Analysis of Biomedical Samples in the Low Attomole Range. Anal. Chem..

[21] Ognibene T.J., Salazar G.A. (2013). Installation of hybrid ion source on the 1-MV LLNL BioAMS spectrometer. Nucl. Instrum. Methods B.

[22] Sacks G.L., Derry L.A., Brenna J.T. (2006). Elemental speciation by parallel elemental and molecular mass spectrometry and peak profile matching. Anal. Chem..

[23] Vogel J.S., Palmblad N.M., Ognibene T., Kabir M.M., Buchholz B.A., Bench G. (2007). Biochemical paths in humans and cells: Frontiers of AMS bioanalysis. Nucl. Instrum. Methods B.

[24] Labrie D., Reid J. (1981). Radiocarbon Dating by Infrared-Laser Spectroscopy—A Feasibility Study. Appl. Phys..

[25] Murnick D.E., Dogru O., Ilkmen E. (2008). Intracavity optogalvanic spectroscopy. An analytical technique for ^14^C analysis with subattomole sensitivity. Anal. Chem..

[26] Galli I., Pastor P.C., Di Lonardo G., Fusina L., Giusfredi G., Mazzotti D., Tamassia F., De Natale P. (2011). The ν_3_ band of ^14^C^16^O_2_ molecule measured by optical-frequency-comb-assisted cavity ring-down spectroscopy. Mol. Phys..

[27] Genoud G., Vainio M., Phillips H., Dean J., Merimaa M. (2015). Radiocarbon dioxide detection based on cavity ring-down spectroscopy and a quantum cascade laser. Opt. Lett..

[28] McCartt A.D., Ognibene T., Bench G., Turteltaub K. (2015). Measurements of Carbon-14 with Cavity Ring-Down Spectroscopy. Nucl. Instrum. Methods Phys. Res. B.

[29] Galli I., Bartalini S., Ballerini R., Barucci M., Cancio P., De Pas M., Giusfredi G., Mazzotti D., Akikusa N., De Natale P. (2016). Spectroscopic detection of radiocarbon dioxide at parts-per-quadrillion sensitivity. Optica.

[30] McCartt A.D., Ognibene T.J., Bench G., Turteltaub K.W. (2016). Quantifying Carbon-14 for Biology Using Cavity Ring-Down Spectroscopy. Anal. Chem..

[31] Fleisher A.J., Long D.A., Liu Q.N., Gameson L., Hodges J.T. (2017). Optical Measurement of Radiocarbon below Unity Fraction Modern by Linear Absorption Spectroscopy. J. Phys. Chem. Lett..

[32] Kratochwil N.A., Dueker S.R., Muri D., Senn C., Yoon H., Yu B.Y., Lee G.H., Dong F., Otteneder M.B. (2018). Nanotracing and cavity-ring down spectroscopy: A new ultrasensitive approach in large molecule drug disposition studies. PLoS ONE.

[33] Taylor R.E. (1987). Dating Techniques in Archaeology and Paleoanthropology. Anal. Chem..

[34] Graven H., Allison C.E., Etheridge D.M., Hammer S., Keeling R.F., Levin I., Meijer H.A.J., Rubino M., Tans P.P., Trudinger C.M. (2017). Compiled records of carbon isotopes in atmospheric CO_2_ for historical simulations in CMIP6. Geosci. Model Dev..

[35] Buchholz B.A., Sarachine M.J., Zermeno P., Ebeler S.E., Takeoka G.R., Winterhalter P. (2011). Establishing Natural Product Content with Natural Radiocarbon Signature. Progress in Authentication of Food and Wine.

[36] Nelson M.A., Ondov J.M., VanDerveer M.C., Buchholz B.A. (2013). Contemporary Fraction of Bis(2-Ethylhexyl) Phthalate in Stilton Cheese by Accelerator Mass Spectrometry. Radiocarbon.

[37] Tong T., Ondov J.M., Buchholz B.A., VanDerveer M.C. (2016). Contemporary carbon content of bis (2-ethylhexyl) phthalate in butter. Food Chem..

[38] Stuiver M., Polach H.A. (1977). Reporting of C-14 Data—Discussion. Radiocarbon.

[39] Hedges R.E.M., Clement J.G., Thomas C.D.L., O’Connell T.C. (2007). Collagen turnover in the adult femoral mid-shaft: Modeled from anthropogenic radiocarbon tracer measurements. Am. J. Phys. Anthropol..

[40] Buchholz B.A., Alkass K., Druid H., Spalding K.L. (2018). Bomb Pulse Radiocarbon Dating of Skeletal Tissues. New Perspect. Forensic Hum. Skelet. Identif..

[41] Shapiro S.D., Endicott S.K., Province M.A., Pierce J.A., Campbell E.J. (1991). Marked Longevity of Human Lung Parenchymal Elastic Fibers Deduced from Prevalence of D-Aspartate and Nuclear-Weapons Related Radiocarbon. J. Clin. Invest..

[42] Heinemeier K.M., Schjerling P., Heinemeier J., Magnusson S.P., Kjaer M. (2013). Lack of tissue renewal in human adult Achilles tendon is revealed by nuclear bomb ^14^C. FASEB J..

[43] Heinemeier K.M., Schjerling P., Heinemeier J., Moller M.B., Krogsgaard M.R., Grum-Schwensen T., Petersen M.M., Kjaer M. (2016). Radiocarbon dating reveals minimal collagen turnover in both healthy and osteoarthritic human cartilage. Sci. Transl. Med..

[44] Lynnerup N., Kjeldsen H., Heegaard S., Jacobsen C., Heinemeier J. (2008). Radiocarbon Dating of the Human Eye Lens Crystallines Reveal Proteins without Carbon Turnover throughout Life. PLoS ONE.

[45] Stewart D.N., Lango J., Nambiar K.P., Falso M.J.S., FitzGerald P.G., Rocke D.M., Hammock B.D., Buchholz B.A. (2013). Carbon turnover in the water-soluble protein of the adult human lens. Mol. Vis..

[46] Lovell M.A., Robertson J.D., Buchholz B.A., Xie C.S., Markesbery W.R. (2002). Use of bomb pulse carbon-14 to age senile plaques and neurofibrillary tangles in Alzheimer’s disease. Neurobiol. Aging.

[47] Goncalves I., Stenstrom K., Skog G., Mattsson S., Nitulescu M., Nilsson J. (2010). Dating components of human atherosclerotic plaques. Eur. Heart J..

[48] Hagg S., Salehpour M., Noori P., Lundstrom J., Possnert G., Takolander R., Konrad P., Rosfors S., Ruusalepp A., Skogsberg J. (2011). Carotid Plaque Age Is a Feature of Plaque Stability Inversely Related to Levels of Plasma Insulin. PLoS ONE.

[49] Etminan N., Dreier R., Buchholz B.A., Beseoglu K., Bruckner P., Matzenauer C., Torner J.C., Brown R.D., Steiger H.J., Haggi D. (2014). Age of Collagen in Intracranial Saccular Aneurysms. Stroke.

[50] Etminan N., Dreier R., Buchholz B.A., Bruckner P., Steiger H.J., Hanggi D., Macdonald R.L. (2013). Exploring the Age of Intracranial Aneurysms Using Carbon Birth Dating Preliminary Results. Stroke.

[51] Spalding K.L., Bhardwaj R.D., Buchholz B.A., Druid H., Frisen J. (2005). Retrospective birth dating of cells in humans. Cell.

[52] Bhardwaj R.D., Curtis M.A., Spalding K.L., Buchholz B.A., Fink D., Bjork-Eriksson T., Nordborg C., Gage F.H., Druid H., Eriksson P.S. (2006). Neocortical neurogenesis in humans is restricted to development. Proc. Natl. Acad. Sci. USA.

[53] Spalding K.L., Bergmann O., Alkass K., Bernard S., Salehpour M., Huttner H.B., Bostrom E., Westerlund I., Vial C., Buchholz B.A. (2013). Dynamics of Hippocampal Neurogenesis in Adult Humans. Cell.

[54] Bergmann O., Liebl J., Bernard S., Alkass K., Yeung M.S., Steier P., Kutschera W., Johnson L., Landen M., Druid H. (2012). The age of olfactory bulb neurons in humans. Neuron.

[55] Yeung M.S., Zdunek S., Bergmann O., Bernard S., Salehpour M., Alkass K., Perl S., Tisdale J., Possnert G., Brundin L. (2014). Dynamics of oligodendrocyte generation and myelination in the human brain. Cell.

[56] Spalding K.L., Arner E., Westermark P.O., Bernard S., Buchholz B.A., Bergmann O., Blomqvist L., Hoffstedt J., Naslund E., Britton T. (2008). Dynamics of fat cell turnover in humans. Nature.

[57] Arner P., Bernard S., Salehpour M., Possnert G., Liebl J., Steier P., Buchholz B.A., Eriksson M., Arner E., Hauner H. (2011). Dynamics of human adipose lipid turnover in health and metabolic disease. Nature.

[58] Bergmann O., Bhardwaj R.D., Bernard S., Zdunek S., Barnabe-Heider F., Walsh S., Zupicich J., Alkass K., Buchholz B.A., Druid H. (2009). Evidence for cardiomyocyte renewal in humans. Science.

[59] Perl S., Kushner J.A., Buchholz B.A., Meeker A.K., Stein G.M., Hsieh M., Kirby M., Pechhold S., Liu E.H., Harlan D.M. (2010). Significant human beta-cell turnover is limited to the first three decades of life as determined by in vivo thymidine analog incorporation and radiocarbon dating. J. Clin. Endocrinol. Metab..

[60] Landsverk O.J., Snir O., Casado R.B., Richter L., Mold J.E., Reu P., Horneland R., Paulsen V., Yaqub S., Aandahl E.M. (2017). Antibody-secreting plasma cells persist for decades in human intestine. J. Exp. Med..

[61] Maze I., Wenderski W., Noh K.M., Bagot R.C., Tzavaras N., Purushothaman I., Elsasser S.J., Guo Y., Ionete C., Hurd Y.L. (2015). Critical Role of Histone Turnover in Neuronal Transcription and Plasticity. Neuron.

[62] Hum N.R., Martin K.A., Malfatti M.A., Haack K., Buchholz B.A., Loots G.G. (2018). Tracking Tumor Colonization in Xenograft Mouse Models Using Accelerator Mass Spectrometry. Sci. Rep..

[63] Orjuela M.A., Liu X., Miller R.L., Warburton D., Tang D., Jobanputra V., Hoepner L., Suen I.H., Diaz-Carreno S., Li Z. (2012). Urinary naphthol metabolites and chromosomal aberrations in 5-year-old children. Cancer Epidemiol. Biomarkers Prev..

[64] Abdo K., Eustic S., McDonald M., Jokinen M., Adkins B., Haseman J. (1992). Naphthalene: A respiratory tract toxicant and carcinogen for mice. Inhal. Toxicol..

[65] North D.W., Abdo K.M., Benson J.M., Dahl A.R., Morris J.B., Renne R., Witschi H. (2008). A review of whole animal bioassays of the carcinogenic potential of naphthalene. Regul. Toxicol. Pharm..

[66] Buchholz B.A., Haack K.W., Sporty J.L., Buckpitt A.R., Morin D. (2010). Free flow electrophoresis separation and AMS quantitation of ^14^C-naphthalene-protein adducts. Nucl. Instrum. Methods B.

[67] Buchholz B.A., Carratt S.A., Kuhn E.A., Collette N.M., Ding X.X., Van Winkle L.S. (2019). Naphthalene DNA adduct formation and tolerance in the lung. Nucl. Instrum. Methods B.

[68] Van Winkle L.S., Kelty J.S., Plopper C.G. (2017). Preparation of Specific Compartments of the Lungs for Pathologic and Biochemical Analysis of Toxicologic Responses. Curr. Protoc. Toxicol..

[69] Morris J.B. (2013). Nasal dosimetry of inspired naphthalene vapor in the male and female B6C3F1 mouse. Toxicology.

[70] Enright H.A., Falso M.J.S., Malfatti M.A., Lao V., Kuhn E.A., Hum N., Shi Y., Sales A.P., Haack K.W., Kulp K.S. (2017). Maternal exposure to an environmentally relevant dose of triclocarban results in perinatal exposure and potential alterations in offspring development in the mouse model. PLoS ONE.

[71] Halden R.U. (2014). On the need and speed of regulating triclosan and triclocarban in the United States. Environ. Sci. Technol..

[72] Coogan M.A., La Point T.W. (2008). Snail bioaccumulation of triclocarban, triclosan, and methyltriclosan in a North Texas, USA, stream affected by wastewater treatment plant runoff. Environ. Toxicol. Chem..

[73] Geiss C., Ruppert K., Heidelbach T., Oehlmann J. (2016). The antimicrobial agents triclocarban and triclosan as potent modulators of reproduction in Potamopyrgus antipodarum (Mollusca: Hydrobiidae). J. Environ. Sci. Health A Tox. Hazard Subst. Environ. Eng..

[74] IARC (2010). Some Non-heterocyclic Polycyclic Aromatic Hydrocarbons and some Related Exposures. Monographs on the Evaluation of Carcinogenic Risks to Humans.

[75] World Health Organization (WHO), International Programme on Chemical Safety (1998). Selected Non-Heterocyclic Polycyclic Aromatic Hydrocarbons.

[76] EPA (2017). Toxicological Review of Benzo[a]pyrene, Integrated Risk Information System, National Center for Environmental Assessment.

[77] Abadin H.G. (2013). The toxicological profile program at ATSDR. J. Environ. Health.

[78] Wheate N.J., Walker S., Craig G.E., Oun R. (2010). The status of platinum anticancer drugs in the clinic and in clinical trials. Dalton Trans..

[79] Dilruba S., Kalayda G.V. (2016). Platinum-based drugs: Past, present and future. Cancer Chemother. Pharmacol..

[80] Kelland L. (2007). The resurgence of platinum-based cancer chemotherapy. Nat. Rev. Cancer.

[81] Wang D., Lippard S.J. (2005). Cellular processing of platinum anticancer drugs. Nat. Rev. Drug Discov..

[82] Galluzzi L., Vitale I., Michels J., Brenner C., Szabadkai G., Harel-Bellan A., Castedo M., Kroemer G. (2014). Systems biology of cisplatin resistance: Past, present and future. Cell Death Dis..

[83] Boocock D.J., Brown K., Gibbs A.H., Sanchez E., Turteltaub K.W., White I.N. (2002). Identification of human CYP forms involved in the activation of tamoxifen and irreversible binding to DNA. Carcinogenesis.

[84] Martin E.A., Brown K., Gaskell M., Al-Azzawi F., Garner R.C., Boocock D.J., Mattock E., Pring D.W., Dingley K., Turteltaub K.W. (2003). Tamoxifen DNA damage detected in human endometrium using accelerator mass spectrometry. Cancer Res..

[85] Hah S.S., Sumbad R.A., de Vere White R.W., Turteltaub K.W., Henderson P.T. (2007). Characterization of oxaliplatin-DNA adduct formation in DNA and differentiation of cancer cell drug sensitivity at microdose concentrations. Chem. Res. Toxicol..

[86] Brown K., Tompkins E.M., Boocock D.J., Martin E.A., Farmer P.B., Turteltaub K.W., Ubick E., Hemingway D., Horner-Glister E., White I.N. (2007). Tamoxifen forms DNA adducts in human colon after administration of a single [^14^C]-labeled therapeutic dose. Cancer Res..

[87] Hah S.S., Henderson P.T., Turteltaub K.W. (2010). Towards biomarker-dependent individualized chemotherapy: Exploring cell-specific differences in oxaliplatin-DNA adduct distribution using accelerator mass spectrometry. Bioorganic Med. Chem. Lett..

[88] Wang S., Zhang H., Malfatti M., de Vere White R., Lara P.N., Turteltaub K., Henderson P., Pan C.X. (2010). Gemcitabine causes minimal modulation of carboplatin-DNA monoadduct formation and repair in bladder cancer cells. Chem. Res. Toxicol..

[89] Henderson P.T., Li T., He M., Zhang H., Malfatti M., Gandara D., Grimminger P.P., Danenberg K.D., Beckett L., de Vere White R.W. (2011). A microdosing approach for characterizing formation and repair of carboplatin-DNA monoadducts and chemoresistance. Int. J. Cancer.

[90] Jiang S., Pan A.W., Lin T.Y., Zhang H., Malfatti M., Turteltaub K., Henderson P.T., Pan C.X. (2015). Paclitaxel Enhances Carboplatin-DNA Adduct Formation and Cytotoxicity. Chem. Res. Toxicol..

[91] Scharadin T.M., Zhang H., Zimmermann M., Wang S., Malfatti M.A., Cimino G.D., Turteltaub K., de Vere White R., Pan C.X., Henderson P.T. (2016). Diagnostic Microdosing Approach to Study Gemcitabine Resistance. Chem. Res. Toxicol..

[92] Wang S., Zhang H., Scharadin T.M., Zimmermann M., Hu B., Pan A.W., Vinall R., Lin T.Y., Cimino G., Chain P. (2016). Molecular Dissection of Induced Platinum Resistance through Functional and Gene Expression Analysis in a Cell Culture Model of Bladder Cancer. PLoS ONE.

[93] Zimmermann M., Wang S.S., Zhang H., Lin T.Y., Malfatti M., Haack K., Ognibene T., Yang H., Airhart S., Turteltaub K.W. (2017). Microdose-Induced Drug-DNA Adducts as Biomarkers of Chemotherapy Resistance in Humans and Mice. Mol. Cancer Ther..

[94] Wang S.S., Zimmermann M., Zhang H., Lin T.Y., Malfatti M., Haack K., Turteltaub K.W., Cimino G.D., de Vere White R., Pan C.X. (2017). A diagnostic microdosing approach to investigate platinum sensitivity in non-small cell lung cancer. Int. J. Cancer.

[95] Wang F., Zhang H., Ma A.H., Yu W., Zimmermann M., Yang J., Hwang S.H., Zhu D., Lin T.Y., Malfatti M. (2017). COX-2/sEH Dual Inhibitor PTUPB Potentiates the Anti-tumor Efficacy of Cisplatin. Mol. Cancer Ther..

[96] Scharadin T.M., Malfatti M.A., Haack K., Turteltaub K.W., Pan C.X., Henderson P.T., Jonas B.A. (2018). Towards predicting AML patient response to 7+3 induction chemotherapy via diagnostic microdosing. Chem. Res. Toxicol..

[97] Eisenhauer E.A., Therasse P., Bogaerts J., Schwartz L.H., Sargent D., Ford R., Dancey J., Arbuck S., Gwyther S., Mooney M. (2009). New response evaluation criteria in solid tumours: Revised RECIST guideline (version 1.1). Eur. J. Cancer.

[98] Rai K.R., Holland J.F., Glidewell O.J., Weinberg V., Brunner K., Obrecht J.P., Preisler H.D., Nawabi I.W., Prager D., Carey R.W. (1981). Treatment of acute myelocytic leukemia: A study by cancer and leukemia group B. Blood.

[99] Lowenberg B., Downing J.R., Burnett A. (1999). Acute myeloid leukemia. N. Engl. J. Med..

[100] Sekeres M.A., Elson P., Kalaycio M.E., Advani A.S., Copelan E.A., Faderl S., Kantarjian H.M., Estey E. (2009). Time from diagnosis to treatment initiation predicts survival in younger, but not older, acute myeloid leukemia patients. Blood.

[101] Juliusson G., Antunovic P., Derolf A., Lehmann S., Mollgard L., Stockelberg D., Tidefelt U., Wahlin A., Hoglund M. (2009). Age and acute myeloid leukemia: Real world data on decision to treat and outcomes from the Swedish Acute Leukemia Registry. Blood.

[102] Kern W., Estey E.H. (2006). High-dose cytosine arabinoside in the treatment of acute myeloid leukemia: Review of three randomized trials. Cancer.

[103] Willemze R., Suciu S., Meloni G., Labar B., Marie J.P., Halkes C.J., Muus P., Mistrik M., Amadori S., Specchia G. (2014). High-dose cytarabine in induction treatment improves the outcome of adult patients younger than age 46 years with acute myeloid leukemia: Results of the EORTC-GIMEMA AML-12 trial. J. Clin. Oncol..

[104] Weick J.K., Kopecky K.J., Appelbaum F.R., Head D.R., Kingsbury L.L., Balcerzak S.P., Bickers J.N., Hynes H.E., Welborn J.L., Simon S.R. (1996). A randomized investigation of high-dose versus standard-dose cytosine arabinoside with daunorubicin in patients with previously untreated acute myeloid leukemia: A Southwest Oncology Group study. Blood.

[105] Bishop J.F., Matthews J.P., Young G.A., Szer J., Gillett A., Joshua D., Bradstock K., Enno A., Wolf M.M., Fox R. (1996). A randomized study of high-dose cytarabine in induction in acute myeloid leukemia. Blood.

[106] Cutts S.M., Swift L.P., Pillay V., Forrest R.A., Nudelman A., Rephaeli A., Phillips D.R. (2007). Activation of clinically used anthracyclines by the formaldehyde-releasing prodrug pivaloyloxymethyl butyrate. Mol. Cancer Ther..

[107] Major P.P., Egan E.M., Beardsley G.P., Minden M.D., Kufe D.W. (1981). Lethality of human myeloblasts correlates with the incorporation of arabinofuranosylcytosine into DNA. Proc. Natl. Acad. Sci. USA.

[108] Kufe D.W., Munroe D., Herrick D., Egan E., Spriggs D. (1984). Effects of 1-beta-D-arabinofuranosylcytosine incorporation on eukaryotic DNA template function. Mol. Pharmacol..

[109] Raza A., Gezer S., Anderson J., Lykins J., Bennett J., Browman G., Goldberg J., Larson R., Vogler R., Preisler H.D. (1992). Relationship of [3H]Ara-C incorporation and response to therapy with high-dose Ara-C in AML patients: A Leukemia Intergroup study. Exp. Hematol..

[110] Gervasoni J.E., Fields S.Z., Krishna S., Baker M.A., Rosado M., Thuraisamy K., Hindenburg A.A., Taub R.N. (1991). Subcellular distribution of daunorubicin in P-glycoprotein-positive and -negative drug-resistant cell lines using laser-assisted confocal microscopy. Cancer Res..

[111] Coley H.M., Amos W.B., Twentyman P.R., Workman P. (1993). Examination by laser scanning confocal fluorescence imaging microscopy of the subcellular localisation of anthracyclines in parent and multidrug resistant cell lines. Br. J. Cancer.

[112] Swift L.P., Rephaeli A., Nudelman A., Phillips D.R., Cutts S.M. (2006). Doxorubicin-DNA adducts induce a non-topoisomerase II-mediated form of cell death. Cancer Res..

[113] Coldwell K.E., Cutts S.M., Ognibene T.J., Henderson P.T., Phillips D.R. (2008). Detection of Adriamycin-DNA adducts by accelerator mass spectrometry at clinically relevant Adriamycin concentrations. Nucleic Acids Res..

[114] Stornetta A., Zimmermann M., Cimino G.D., Henderson P.T., Sturla S.J. (2016). DNA Adducts from Anticancer Drugs as Candidate Predictive Markers for Precision Medicine. Chem. Res. Toxicol..

[115] Swart P., Lozac’h F., Simon M., van Duijn E., Vaes W.H. (2016). The impact of early human data on clinical development: There is time to win. Drug Discov. Today.

[116] Morcos P.N., Yu L., Bogman K., Sato M., Katsuki H., Kawashima K., Moore D.J., Whayman M., Nieforth K., Heinig K. (2017). Absorption, distribution, metabolism and excretion (ADME) of the ALK inhibitor alectinib: Results from an absolute bioavailability and mass balance study in healthy subjects. Xenobiotica.

[117] Husser C., Pahler A., Seymour M., Kuhlmann O., Schadt S., Zell M. (2018). Profiling of dalcetrapib metabolites in human plasma by accelerator mass spectrometry and investigation of the free phenothiol by derivatisation with methylacrylate. J. Pharm. Biomed. Anal..

[118] Schadt S., Bister B., Chowdhury S.K., Funk C., Hop C., Humphreys W.G., Igarashi F., James A.D., Kagan M., Khojasteh S.C. (2018). A Decade in the MIST: Learnings from Investigations of Drug Metabolites in Drug Development under the “Metabolites in Safety Testing” Regulatory Guidance. Drug Metab. Dispos..

